# Gastroesophageal Reflux Disease Symptoms and Their Association With Dyspepsia Severity: A Questionnaire-Based Cross-Sectional Study

**DOI:** 10.7759/cureus.98248

**Published:** 2025-12-01

**Authors:** Samraiz Nafees, Asir Sharif, Khaled Zamari, Mohamed Abdelfattah, Ali Javeed, Vikram Kumar, Nuwanthi Upeka Wanigasinghe, Juweria Shahrukh Effendi, Melna Mathew, Sashank Manda, Sanath

**Affiliations:** 1 Internal Medicine, Services Institute of Medical Sciences, Lahore, PAK; 2 Internal Medicine, York and Scarborough Teaching Hospitals NHS Foundation Trust, Scarborough, GBR; 3 General Practice, Gloucestershire Hospitals NHS Foundation Trust, Bath, GBR; 4 Internal Medicine, Scarborough General Hospital, Scarborough, GBR; 5 Internal Medicine, Liaquat University of Medical and Health Sciences, Jamshoro, PAK; 6 Internal Medicine, Hamdard College of Medicine and Dentistry, Karachi, PAK; 7 Medicine, Scarborough General Hospital, Scarborough, GBR

**Keywords:** dyspepsia, gastroesophageal reflux disease, gastrointestinal disorders, gerd-q, sf-ldq

## Abstract

Background: Gastroesophageal reflux disease (GERD) and dyspepsia are typical gastrointestinal disorders that may overlap, making it difficult to diagnose and treat them. There is little local information on their connection in Pakistan. This study aimed to determine the connection between GERD symptoms and the severity of dyspepsia in adults.

Methods: The study was a cross-sectional study in the outpatient clinics of hospitals in Karachi and Lahore. Convenience sampling was used to recruit 310 adults with dyspeptic symptoms. The data were collected using the Gastroesophageal Reflux Disease Questionnaire (GERD-Q) and the Short-Form Leeds Dyspepsia Questionnaire (SF-LDQ), along with demographic and clinical variables. The study was conducted between August and September 2025 at the outpatient clinics of hospitals in Karachi and Lahore. Data were analyzed statistically using SPSS v.26, and descriptive statistics, Spearman correlation, Mann-Whitney U tests, Kruskal-Wallis tests, chi-square tests, and multiple linear regression models were employed.

Results: The average GERD-Q score was 15.69 ± 2.28, and the average SF-LDQ score was 14.60 ± 1.67. The GERD-Q and SF-LDQ scores were positively correlated (p = 0.001). Females scored significantly higher than males on both the GERD-Q and SF-LDQ (p< 0.05). Higher scores on the GERD-Q were found in older individuals, while the severity of dyspepsia was more pronounced among younger subjects (p< 0.05). Regression analysis revealed that GERD symptoms, female gender, a family history of gastrointestinal disorders, and a chronic illness are significant predictors of dyspepsia severity (p < 0.01).

Conclusion: More severe dyspepsia was very closely related to GERD symptomatology in this Pakistani sample. Symptom burden was significantly associated with gender, age, family history, and chronic illness. These findings give insight into the area of overlap between GERD and dyspepsia, indicating the need for concurrent diagnostic and therapeutic approaches in assessing such populations within local validity.

## Introduction

Gastroesophageal reflux disease (GERD) is a common condition with a prevalence of about 20% in adults from affluent countries and severe ramifications in the quality of life [1]. GERD is a significant health issue, but the clinical manifestation is challenging to diagnose [2]. Typical symptoms of GERD include heartburn or regurgitation; common effects of GERD are esophagitis, strictures, Barrett's esophagus, and adenocarcinoma [3].

The systematic reviews show that the prevalence of GERD is increasing worldwide, with estimates of 18-28% in North America, 9-26% in Europe, and up to 33% in the Middle East, and the much lower prevalence (less than 10%) in East Asia. This reflects an increasing disease burden, accompanied by significant geographic diversity [4]. High cholesterol and smoking have been proposed to be independent risk factors of GERD. It is also associated with a poor quality of life, particularly in terms of physical functioning, regardless of symptom severity and frequency [5].

Dyspepsia is a widespread condition that can be categorized into postprandial distress, epigastric pain, or combined subtypes. It is caused by dysfunctional motility, visceral hypersensitivity, or mucosal inflammation, and frequently by a remarkably high level of quality-of-life impairment [6,7]. Affecting approximately 25% of the population each year, dyspepsia is often similar to the symptoms of reflux disease and irritable bowel syndrome (IBS), so it is difficult to diagnose. Symptom subgroups demonstrate a significant overlap and limited diagnostic utility; however, alarm features such as weight loss, vomiting, or bleeding should be investigated further [8].

GERD often coexists with functional gastrointestinal disorders, most prominently functional dyspepsia and IBS [9]. Symptoms of a dyspeptic nature are common in patients with GERD, including bloating, belching, early satiety, and epigastric pain. These symptoms tend to be more common in nonerosive disease and are a significant burden on quality of life, with variable responses to proton pump inhibitor therapy [10].

This study aims to assess the severity of GERD symptoms and their correlation with dyspepsia severity in a cross-sectional sample using a questionnaire. The findings can be used to enhance recognition, personalized management, and patient outcomes by identifying patterns of overlap.

Rationale

Both GERD and dyspepsia are prevalent conditions that often co-occur; therefore, distinguishing between them can be challenging in routine practice. The relationship between reflux symptoms and dyspepsia severity has not been previously studied in the country, with limited local data available on the topic. Because other countries have different healthcare systems and patient populations, it is necessary to produce evidence representative of trends observed in Pakistan, rather than relying on data from other global populations. Previous local studies have primarily focused on the prevalence and lifestyle determinants of GERD in specific populations, such as medical students, without examining its overlap with dyspepsia or other functional gastrointestinal symptoms [11]. This highlights the need for locally relevant research addressing both disorders concurrently.

In Pakistan, lifestyle habits such as frequent consumption of spicy or acidic foods, irregular meal patterns, high intake of caffeine or carbonated beverages, and large meals before bedtime have been associated with an increased prevalence of gastroesophageal reflux disease [11]. Most people do not seek treatment until symptoms are severe, which complicates diagnosis and treatment. Investigating the relationship between GERD symptoms and dyspepsia levels in a local sample can provide information directly applicable to clinical practice in Pakistan and potentially aid in the development of more targeted management interventions.

Objectives

The primary aim of this study was to evaluate the correlation between gastroesophageal reflux disease (GERD) symptoms and dyspepsia severity. We hypothesized that higher GERD symptom scores would be associated with greater dyspepsia severity among participants. Secondary objectives included determining the prevalence of GERD symptoms, assessing dyspepsia severity using a validated questionnaire, and investigating the relationships between demographic variables (age, gender) and GERD symptoms and dyspepsia severity. This study addresses a gap in regional data, as limited information exists on the prevalence and severity of GERD and dyspepsia in Pakistan, providing insights into symptom patterns and associated risk factors in the local population.

## Materials and methods

Methodology and approach

In the current study, a cross-sectional design was employed to investigate the relationship between symptoms of GERD and dyspepsia in Pakistani adults. The participants were identified in the outpatient clinics of the chosen hospitals in Karachi and Lahore. The structured questionnaire-based instrument was used to collect data on demographics and lifestyle, symptoms of GERD, and severity of dyspepsia. The study was conducted between August and September 2025 at the outpatient clinics of hospitals in Karachi and Lahore.

The trained research assistants contacted the patients, explained the purpose of the study, and provided them with ample opportunity to ask questions before obtaining informed consent. The participants were also assured of anonymity and told that they had the right to withdraw at any time. This methodology facilitated culturally suitable interactions, reduced the discomfort of the participants, and ensured the quality and reliability of the data collection.

Recruitment and sample

The study assumed a prevalence (p) of 0.5 and an infinite population, as no local data were available. The 95% confidence interval (Z = 1.96) and margin of error (0.05) were used to achieve this [12]. Convenience sampling was used to recruit adults from outpatient hospitals in Karachi and Lahore. Convenience sampling was used due to limited access to a complete sampling frame and logistical constraints, which are common in hospital-based epidemiological studies. This approach allowed efficient recruitment, though it may limit generalizability. Among the 350 people contacted, 40 were deemed ineligible or refused to participate. The remaining 310 respondents (88.6%) provided informed consent, completed the study questionnaire, and were included in the final analysis. The convenience sampling technique was found to be an efficient means of recruiting the participants at the right time and making the resources manageable. Nevertheless, it might have led to selection bias, and the results cannot be generalized to the general Pakistani population.

Eligibility criteria

The participants in the study were adults aged 18 years and above who presented at the outpatient clinic of the selected hospitals in Karachi and Lahore with complaints of dyspepsia. Inclusion in the study was limited to individuals who agreed to complete the questionnaire. Patients with a past diagnosis of peptic ulcer disease, gastrointestinal malignancy, or a history of gastric surgery were excluded. Individuals who have taken proton pump inhibitors, H2 receptor blockers, or other medications likely to influence gastrointestinal symptoms for an extended period are not eligible. Also, pregnant and lactating women, as well as patients who had not given or were not able to provide informed consent, were excluded from the study.

Instruments and procedures

In this study, data were collected using a structured questionnaire, which was divided into three major parts: demographic data, the Gastroesophageal Reflux Disease Questionnaire (GERD-Q), and the short-form Leeds Dyspepsia Questionnaire (SF-LDQ). The People section provided various details, including age, gender, smoking status, and other relevant information.

The demographic section was used to gather crucial information about the participants, including age, gender, smoking status, educational level, and any relevant medical history. Such information aided in contextualising the findings and enabled the subgroup analysis to evaluate possible relationships between demographic variables and the prevalence or severity of gastrointestinal symptoms (Appendix A).

The GERD-Q assessed how often and how many people experience symptoms related to gastroesophageal reflux disease (GERD). The instrument, developed by Jones et al. in 2009, consists of six items with response options scored on a four-point Likert scale (0-3). The total score ranges from 0 to 18. If the score is eight or higher, there is a high probability of having GERD. The GERD-Q addresses key symptoms such as heartburn, acid reflux, and sleep disturbances, and also includes additional medications. The instrument is brief, self-report, and pretty reliable (alpha 0.70-0.85) in various populations (Appendix B) [13]. The Gastroesophageal Reflux Disease Questionnaire (GERD-Q) was administered with formal permission obtained from Professor Janice Rymer (on behalf of Professor Jones, King's College London).

In 2007, Fraser et al. published the Short-Form Leeds Dyspepsia Questionnaire (SF-LDQ), a simplified version of the LDQ. It consists of four items that measure how often and how bad dyspepsia symptoms, such as epigastric pain, bloating, nausea, and early satiety, are. On a scale of 1 to 5, the higher the number, the worse the symptoms. The total score indicates the extent of the burden caused by dyspepsia. The SF-LDQ has a Cronbach's alpha of above 0.80, with good internal consistency. It is considered to be both valid and reliable, and can be used for research and practice (Appendix C) [14]. The Short-Form Leeds Dyspepsia Questionnaire was utilised with formal approval granted by Professor Alex Ford.

Potential participants were informed about the study's objective before enrollment, and they provided written informed consent after being trained by research assistants. The researcher obtained permission from the respondents before handing over the structured questionnaire. They filled it out individually or with the help of a research assistant, depending on their literacy levels. Research assistants ensured that participants understood and paid attention to the instructions, made sure the instructions were culturally sensitive, and addressed any questions that arose during the process. They verified all the completed questionnaires and stored them in a secure location for use during analysis.

Statistical plan

IBM SPSS Statistics version 26 has been used for data analysis. Data were tested for normality using the Shapiro-Wilk test, and as the distributions were non-normal, non-parametric tests were applied where appropriate. The frequencies and percentages are proving helpful in summarizing the descriptive statistics of the demographic variables of the research participants. Histograms were used to analyze the distributions of GERD-Q and SF-LDQ scores. Spearman correlation was performed to assess the association between the GERD-Q and SF-LDQ scores. The Mann-Whitney U test was used to analyze gender differences in GERD-Q and SF-LDQ, while the Kruskal-Wallis test evaluated the differences between age groups. We used multiple linear regression analysis to identify the factors that predict the severity of dyspepsia (SF-LDQ), the dependent variable, in relation to GERD-Q scores, age, sex, family history of gastrointestinal disorders, and chronic illness. The model’s explanatory power was assessed using R² and Adjusted R² values, which are reported in the results section. To assess the effect of family history of gastrointestinal disorders on gender, the chi-square test of independence was applied. Cramer V is the variable of effect size. All statistical tests were two-tailed, with a significant value of p< 0.05. Smoking status was recorded for descriptive purposes only and was not included in the inferential analyses because preliminary testing showed no meaningful variation in symptom scores across smoking categories.

Ethical standards

The study was conducted in accordance with ethical guidelines on human research. The Institute of Liaquat University of Medical and Health Sciences approved the survey through the IRB (072/LUMHS/IRB/25). The approval was necessary to ensure adherence to the study's principles of autonomy, beneficence, non-maleficence, and confidentiality. Participants were entirely voluntary and were assured of the freedom to withdraw at any time without any adverse consequences.

All personal information was considered confidential and intended for use in the research. The questionnaires were anonymised and stored in a secure location that was inaccessible to all third parties. The missing or incomplete answers were handled with great care to avoid compromising the validity and reliability of the findings. Particularly, missing values that were not essential were addressed through pairwise deletion, and participants who had answered fewer than 20% of the questionnaire were excluded from the analysis. This was done to maintain the integrity of the study and protect the rights, dignity, and privacy of the participants.

## Results

Table 1 shows the demographic features of the 310 participants. The majority were aged 18-25 years (N = 180, 58%), 26-35 years (N = 80, 26%), 36-45 years (N = 30, 10%), 46-55 years (N = 12, 4%), and 56 years and above (N = 8, 2%). The number of females was 180 (58%), and that of males was 130 (42%). On marital status, 151 (49%) were married, 88 (28%) were divorced or widowed, and 71(23%) were single. Regarding education, 101 respondents (33%) had a primary or middle school education, 75 (24%) had a high school education, 71 (23%) had no formal education, 46 (15%) had an undergraduate degree, and 17 (5%) had a postgraduate or higher education. In terms of occupation, 102 (33) were homemakers, 98 (32%) employed, 64 (21) students, and 46 (15) unemployed. Smoking status indicated that 132 (43%) participants were current smokers, 110 (35%) were former smokers, and 68 (22%) were never smokers. 150 (48%) had a family history of gastrointestinal disorders, 120 (39%) did not, and 40 (13%) did not know. On chronic illness, 102 of the participants (33%) reported having hypertension, 80 (26%) diabetes, 35 (11%) cardiovascular disease, 9 (3%) other illness, and 84 (27%) none.

**Table 1:** Demographic characteristics of participants (N = 310). f: frequency, %: percentage; Values are presented as N (%), N = 310; No statistical comparisons were performed for demographic variables in this table.

Variable	f	%
Age	-	-
18–25 years	180	58
26–35 years	80	26
36–45 years	30	10
46–55 years	12	4
56 years and above	8	2
Gender	-	-
Female	180	58
Male	130	42
Marital status	-	-
Single	71	23
Married	151	49
Divorced/widowed	88	28
Educational level	-	-
No formal education	71	23
Primary/middle school	101	33
High school	75	24
Undergraduate	46	15
Postgraduate or above	17	5
Occupation	-	-
Student	64	21
Employed	98	32
Homemaker	102	33
Unemployed	46	15
Smoking status	-	-
Never smoked	68	22
Former smoker	110	35
Current smoker	132	43
Family history of GI disorders (GERD/dyspepsia/peptic ulcer)	-	-
Yes	150	48
No	120	39
Not sure	40	13
Chronic Illness	-	-
None	84	27
Hypertension	102	33
Diabetes	80	26
Cardiovascular disease	35	11
Other	9	3

**Figure 1:**
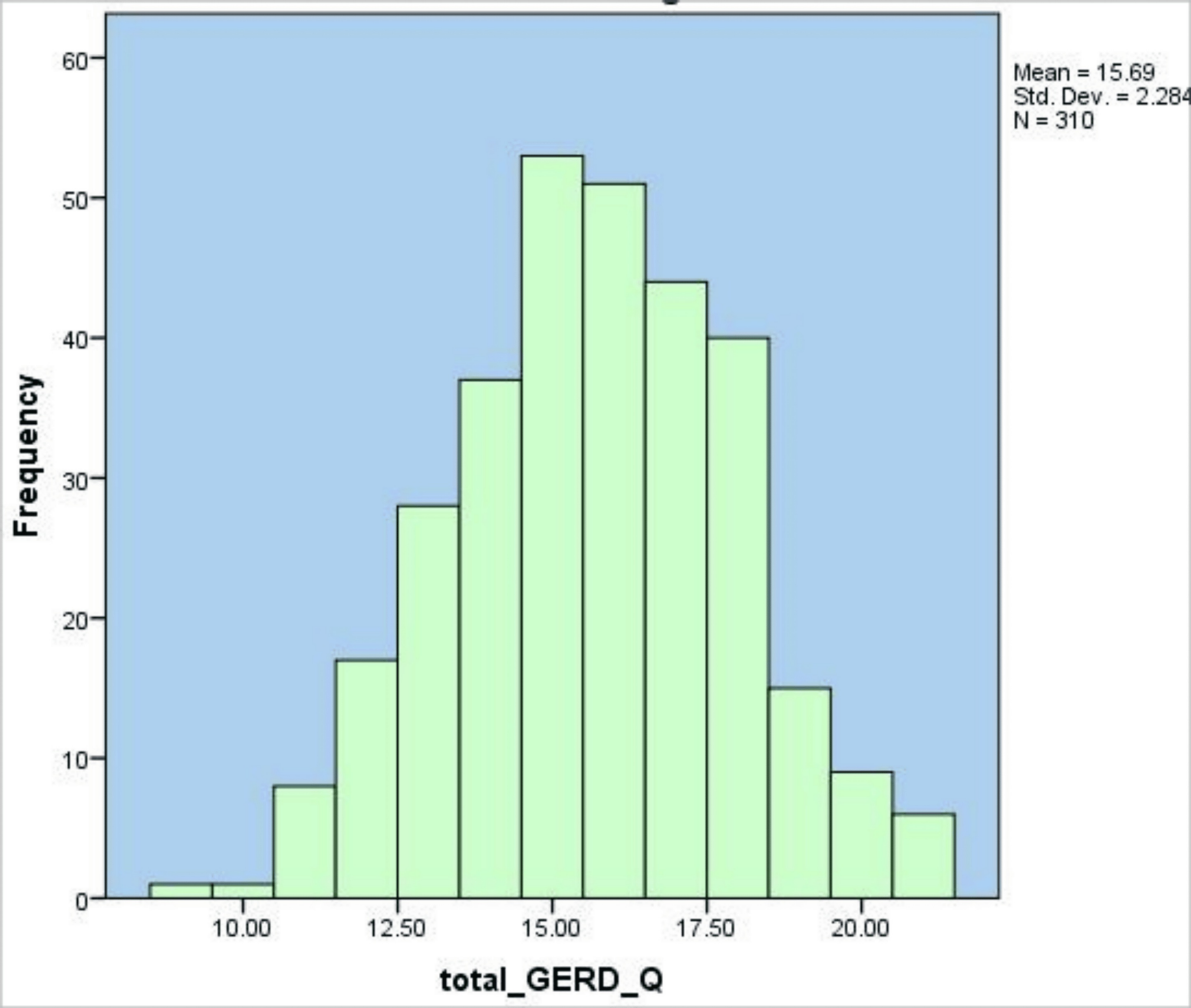
Histogram of Gastroesophageal Reflux Disease Questionnaire (GERD-Q) total scores among participants (N = 310). The histogram displays the distribution of total GERD-Q for 310 participants. Scores ranged from approximately 9 to 21, with a mean of 15.69 (SD = 2.28). The distribution is approximately normal, with the highest frequency of scores falling between 14 and 16. The histogram illustrates that most participants scored within one standard deviation of the mean, indicating moderate variability in GERD-related symptom reporting.

Figure 1 demonstrates the distribution of GERD-Q total scores in the case of 310 participants. The histogram indicates an approximately normal distribution, with the majority of scores falling between 13 and 18. The average result was 15.69 (SD = 2.28), suggesting that most participants experienced moderate GERD-related symptoms, with few respondents at the extremes of low (<12) or high (>20) levels.

**Figure 2:**
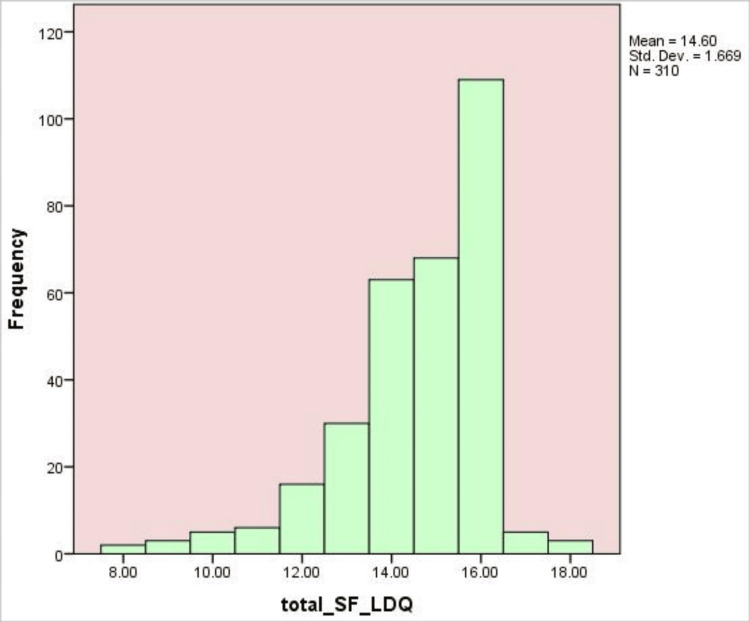
Histogram of Short-Form Leeds Dyspepsia Questionnaire (SF-LDQ) total scores among participants (N = 310). The histogram shows the distribution of total scores on the SF-LDQ for 310 participants. Scores ranged from approximately 8 to 18, with a mean of 14.60 (SD = 1.67). The distribution is moderately skewed toward higher values, with the greatest frequency of scores between 14 and 16. Most participants scored above the mean, indicating that higher dyspepsia symptom burden was common in this sample. The clustering of scores suggests relatively low variability in responses compared with a normal distribution.

Figure 2 shows a histogram of overall scores on the SF-LDQ in 310 participants. The distribution is skewed to the right, with most participants scoring 14 to 16, implying a cluster of severity of the dyspeptic symptoms. The mean score are 14.60 and a standard deviation of 1.669, indicating minimal variation in responses. The highest frequency is observed at a score of approximately 15, with the number of participants scoring lower being fewer, indicating that the majority of participants had moderate to high levels of dyspepsia symptoms.

Table 2 shows the correlation between the GERD-Q and SF-LDQ scores. The GERD-Q and SF-LDQ scores showed a statistically significant positive correlation (r = 0.265, t(308) = 4.80, p< 0.001), indicating a weak but significant association. This suggests that while the relationship is statistically significant, its clinical relevance may be limited.

**Table 2:** Spearman correlation between Gastroesophageal Reflux Disease Questionnaire (GERD-Q) and Short-Form Leeds Dyspepsia Questionnaire (SF-LDQ) scores (N = 310). All values are Spearman's rho (ρ) with corresponding t statistics (df = 308) and two-tailed significance levels; p< 0.001** indicates statistical significance.

Variables	Gastroesophageal Reflux Disease Questionnaire (GERD-Q)	Short-Form Leeds Dyspepsia Questionnaire (SF-LDQ)
Gastroesophageal Reflux Disease Questionnaire (GERD-Q)	–	Spearman’s ρ = 0.265, t(308) = 4.80, p <0.001^**^
Short-Form Leeds Dyspepsia Questionnaire (SF-LDQ)	–	–

Table 3 shows the outcome of the Mann-Whitney U test between genders regarding the GERD-Q and SF-LDQ scores of 310 respondents. Women were found to have better mean ranks in both GERD-Q (163.20 vs. 145.20) and SF-LDQ (167.90 vs. 140.80) compared to men, indicating that they report more severe gastroesophageal reflux and dyspepsia symptoms. Such differences were found to be statistically significant (p< 0.05), indicating a significant gender difference in the self-reported occurrence or intensity of upper gastrointestinal symptoms.

**Table 3:** Mann–Whitney U test results for gender differences in Gastroesophageal Reflux Disease Questionnaire (GERD-Q) and Short-Form Leeds Dyspepsia Questionnaire (SF-LDQ) scores (N = 310). N: number of participants; Results from Mann–Whitney U tests comparing men and women on GERD-Q and SF-LDQ; All comparisons were statistically significant at *p< 0.05.

Outcome	Gender	N	Mean rank	Sum of ranks
Gastroesophageal Reflux Disease Questionnaire (GERD-Q)	Male	130	145.2	18,600.00
	Female	180	163.2	29,605.00
Short-Form Leeds Dyspepsia Questionnaire (SF-LDQ)	Male	130	140.8	18,304.00
	Female	180	167.9	29,901.00

Table 4 provides a summary of the Mann-Whitney U test statistics on the scores of the GERD-Q and SF-LDQ between male and female participants. The findings showed statistically significant differences in both GERD-Q (U = 10,085.00, Z = -2.07, p = 0.038) and SF-LDQ scores (U = 9,789.00, Z = -2.45, p = 0.014), showing that the female group had higher symptom severity than the male one. These results support the identified gender difference in upper GI symptoms, where women had a greater reflux and dyspeptic symptom burden.

**Table 4:** Test statistics and comparison of Gastroesophageal Reflux Disease Questionnaire (GERD-Q) and Short-Form Leeds Dyspepsia Questionnaire (SF-LDQ) scores between male and female participants using the Mann–Whitney U test. Results from Mann–Whitney U tests comparing men and women on GERD-Q and SF-LDQ; All comparisons were statistically significant at *p< 0.05.

Test statistics	Mann-Whitney U	Wilcoxon W	Z	p
Gastroesophageal Reflux Disease Questionnaire (GERD-Q)	10,085.000	18,600.000	–2.07	0.038^*^
Short-Form Leeds Dyspepsia Questionnaire (SF-LDQ)	9,789.000	18,304.000	-2.45	0.014^*^

Table 5 presents the Kruskal-Wallis test results for GERD-Q and SF-LDQ scores across age groups in 310 participants. GERD-Q mean ranks increased with age (145.20 in 18-25 years to 182.6 in 56+ years; H = 10.82, p = 0.029), indicating greater reflux severity in older adults. Post-hoc analysis showed significantly higher GERD-Q scores in the 46-55 (p = 0.021) and 56+ (p = 0.037) groups compared with the youngest group. In contrast, SF-LDQ scores decreased with age (168.90 in 18-25 years to 136.30 in 56+ years; H = 11.74, p = 0.019), suggesting more dyspeptic symptoms among younger participants (p = 0.015-0.022). This pattern may reflect physiological and lifestyle factors: GERD tends to become more pronounced with age due to reduced lower oesophageal sphincter tone, comorbidities, and medication use, whereas younger adults may experience more dyspeptic symptoms linked to stress, irregular eating habits, and consumption of spicy or fast foods.

**Table 5:** Kruskal–Wallis test results comparing Gastroesophageal Reflux Disease Questionnaire (GERD-Q) symptoms and Short-Form Leeds Dyspepsia Questionnaire (SF-LDQ) across age groups (N = 310). N: number of participants; Results are from Kruskal–Wallis tests; Effect sizes (r) were calculated as √χ2/N; *p< 0.05.

Outcome	Age Group	N	Mean rank	H (df)	p	Post-hoc comparisons (Dunn–Bonferroni)
Gastroesophageal Reflux Disease Questionnaire (GERD-Q)	18–25 years	180	145.20	10.82 (4)	0.029*	46–55 years > 18–25 years, (p = 0.021); 56+ years > 18–25 years, (p = 0.037)
-	26–35 years	80	150.50	-	-	-
-	36–45 years	30	165.80	-	-	-
-	46–55 years	12	176.40	-	-	-
-	56 years and above	8	182.60	-	-	-
Short-Form Leeds Dyspepsia Questionnaire (SF-LDQ)	18–25 years	180	168.90	11.74 (4)	0.019*	18–25 years > 46–55 years, (p = 0.015); 18–25 years > 56+ years, (p = 0.022)
-	26–35 years	80	162.40	-	-	-
-	36–45 years	30	150.10	-	-	-
-	46–55 years	12	140.50	-	-	-
-	56 years and above	8	136.30	-	-	-

Table 6 presents the outcomes of the Kruskal-Wallis H test, which compares the GERD-Q and SF-LDQ scores of various age groups. The analysis results showed that both GERD-Q (χ² (4) = 10.85, p = 0.028, r = 0.19) and SF-LDQ (χ² (4) = 12.42, p = 0.015, r = 0.20) exhibited significant differences based on age. The small-to-moderate effect sizes indicate that symptom differences across age groups were significant but relatively small. All in all, these findings suggest that both GERD and dyspeptic symptoms differ significantly with age.

**Table 6:** Test statistics and differences in Gastroesophageal Reflux Disease Questionnaire (GERD-Q) and Short-Form Leeds Dyspepsia Questionnaire (SF-LDQ) scores across age groups based on the Kruskal–Wallis H test. Results are from Kruskal–Wallis tests; Effect sizes (r) were calculated as √χ2/N; *p< 0.05.

Test statistics	x^2^(df=4)	p	r
Gastroesophageal Reflux Disease Questionnaire (GERD-Q)	10.85	0.028^*^	0.19
Short-Form Leeds Dyspepsia Questionnaire (SF-LDQ)	12.42	0.015^*^	0.20

Table 7 is a multiple regression model of dyspepsia severity (SF-LDQ) based on the GERD symptoms, age, gender, family history of gastrointestinal disorders, and chronic illness. The general model showed that all predictors had a significant contribution to SF-LDQ scores. In particular, the GERD-Q scores showed a higher correlation with the severity of dyspepsia (B = 0.120, 0.165, t = 3.43, p = 0.001). The relationship between age and dyspepsia was negative (B = -0.980, SE = 0.145, t = -2.33, p = 0.020), indicating that older participants had a lower level of dyspepsia. Gender (women) was predicted to be higher in SF-LDQ scores (B = 1.250, β = 0.175, t = 3.29, p = 0.001), family history of gastrointestinal disorders (B = 0.420, 8 = 0.160, t = 3.50, p = 0.001) and having any known chronic illness in the family (B = 0.580, 8 = 0.170 These results indicate that symptoms of GERD, demographic characteristics, family history, and chronic disease are significant predictors of the severity of dyspepsia among the participants.

**Table 7:** Multiple regression predicting dyspepsia severity from gastroesophageal reflux disease (GERD) symptoms, age, gender, family history, and chronic illness (N = 310). Note. R^2^: 0.38, Adjusted R^2^: 0.36, F(5,324): 39.50, **p< 0.001. B: unstandardized coefficients; SE: standard errors; β: standardized coefficients; t: test statistics; p: significance levels; Cl: 95% confidence intervals; LL: lower limit; UL: upper limit; *p< 0.05, **p< 0.01 are considered significant.

Predictor	B	SE	β	t	p	95% CI, LL	95% CI, UL
Constants	8.210	1.520	–	5.40	<0.001^**^	5.22	11.20
GERD-Q	0.120	0.035	0.165	3.43	0.001^**^	0.051	0.189
Age	–0.980	0.420	–0.145	–2.33	0.020^*^	–1.81	–0.15
Gender (Female = 1)	1.250	0.380	0.175	3.29	0.001^**^	0.50	2.00
Family history of GI disorders	0.420	0.120	0.160	3.50	<0.001^**^	0.18	0.66
Any known chronic illness	0.580	0.180	0.170	3.22	0.002^**^	0.23	0.93

Table 8 demonstrates the correlation between gender and family history and gastrointestinal disorders. In males, 70 (53.8%) had a positive family history, 40 (30.8%) had no family history, and 20 (15.4%) were unaware of their family history. In females, 80 participants (44.4%) had a positive family history, 80 (44.4%) had a negative one, and 20 (11.1%) were unsure. The chi-square test revealed a statistically significant relationship between gender and a family history of gastrointestinal disorders (χ² = 6.09, df = 2, p = 0.048). Still, the effect size (Cramer's V = 0.14) is small, indicating that the distribution of family history of gastrointestinal disorders is slightly different between male and female participants.

**Table 8:** Association between gender and family history of gastrointestinal disorders (N = 310). Note. N: frequency; %: percentage; df: degree of freedom; x^2^:chi-square statistics; p: level of significance; p-values calculated using the chi-square test; χ² (2, N = 310) = 6.09, p< 0.05*.

Family history of GI disorders	Male N (%)	Female N (%)	Total N(%)	x^2^(df=2)	p	Cramer's V
Yes	70 (53.8%)	80 (44.4%)	150 (48.4%)	-	-	-
No	40 (30.8%)	80 (44.4%)	120 (38.7%)	-	-	-
Not sure	20 (15.4%)	20 (11.1%)	40 (12.9%)	-	-	-
Total	130 (100%)	180 (100%)	310 (100%)	6.09	0.048^*^	0.14

## Discussion

In this study, the relationship between gastro-esophageal reflux disease (GERD) symptoms and the severity of dyspeptic symptoms was assessed in a Pakistani out-patient population. We observed a weak but statistically significant positive correlation between GERD-Q and SF-LDQ scores, indicating that, on average, more reflux symptoms were slightly associated with more dyspeptic symptoms. This is in keeping with previous reports showing that although GERD and functional dyspepsia are considered different concepts, they often overlap symptomatically and diagnostically, thus making them difficult to separate clearly in clinical practice [15].

Women in our cohort scored higher on the GERD-Q and SF-LDQ, suggesting a greater symptom burden. These results are consistent with the earlier reports according to which women are more likely to experience and report reflux and dyspeptic symptoms, more intense and lower quality of life [16,17]. These differences may be attributed to biological, hormonal, and psychosocial factors influencing symptom perception. However, this finding should be interpreted with caution, as the sample included a higher proportion of females (58%), which may have contributed to the observed gender difference.

In our research, the GERD-Q scores were significantly correlated with age, indicating that older participants reported more reflux symptoms. The same age-related patterns have been reported in extensive population-based surveys, which also suggest a gradual increase in the prevalence of reflux symptoms with age [18]. Conversely, we found that dyspepsia levels decreased with age, with younger individuals reporting stronger symptoms. This result aligns with previous studies on the community, which have also observed a reduction in symptom frequency with age [19]. These contrasting trends may reflect physiological and lifestyle differences: older adults may experience more reflux due to declining esophageal sphincter function, comorbidities, and medication use, whereas younger individuals may be more prone to dyspeptic symptoms related to stress, irregular eating habits, and higher intake of spicy or fast foods.

The symptoms of GERD were significantly associated with the severity of dyspepsia, reflecting the overlap between the two conditions [15]. Further, women tended to have more severe dyspepsia, which complies with the findings that dyspepsia is disproportionately prevalent in women [17]. Additionally, the severity of dyspepsia decreases with age, as earlier studies have indicated that the prevalence of dyspepsia symptoms declines with age [19]. Our research showed that a positive family history of GI disorders was associated with higher dyspeptic severity, which aligns with past literature that family predisposition is closely linked with functional gastrointestinal symptoms [20]. Likewise, the chronic illness was associated with increased dyspeptic severity, corroborating earlier results that comorbidities and overlapping disorders increase the gastrointestinal symptom load and quality of life [21].

Interestingly, men in our study had higher chances of reporting a family history of gastrointestinal disorders compared to their female counterparts. Although previous studies have established close relations between family history and functional gastrointestinal disorders, gender variations have not been put into perspective [22]. Our results thus add new knowledge to the existing understanding of gender-related variations in the reporting or awareness of familial GI conditions.

Study limitations and implications for future research

This study has several limitations. To begin with, the cross-sectional design does not allow for making causal inferences, thus constraining the ability to determine the directionality of the relationship between GERD symptoms and the severity of dyspepsia. Second, as participants were recruited from hospital outpatient clinics, there is a potential sampling bias toward individuals with more severe symptoms. In addition, the use of self-reported questionnaires may introduce recall bias and misclassification, which could affect the accuracy of reported symptom prevalence. Third, the convenience sampling technique used in outpatient clinics in Karachi and Lahore may have introduced selection bias, thereby limiting the study's applicability to the entire Pakistani population. Fourth, the relatively short data collection period (August-September 2025) may not have captured possible seasonal variations in gastrointestinal symptoms, as factors such as dietary habits, temperature, and lifestyle changes across seasons could influence symptom presentation. Fifth, lifestyle issues, including diet and stress levels, were evaluated indirectly without the use of objective measures. Additionally, smoking was assessed only descriptively and not incorporated into the analytical models, which might limit the interpretation of its contribution to GERD and dyspepsia severity. The gender imbalance in the sample, with women representing 58% of participants, may also have introduced a source of bias, as women generally report upper gastrointestinal symptoms more frequently and intensely, potentially exaggerating gender-related associations. Sixth, both GERD-Q and SF-LDQ assess overlapping upper gastrointestinal symptoms, which may have inflated correlations between the two measures, representing a methodological limitation. Furthermore, regional diagnostic gaps in Pakistan limit the contextualization of our findings, as limited local data exist on symptom overlap between GERD and dyspepsia; this restricts broader regional comparison despite the study’s contribution to filling this gap. Lastly, the lack of endoscopic or pH-metric confirmation suggests that symptom-based diagnoses may not be entirely complete in describing the underlying pathophysiology.

Future studies are recommended to utilize longitudinal study designs to investigate the relationship between GERD and dyspepsia symptoms over time. Recruitment should be expanded to encompass a broader range of geographical and socioeconomic categories in Pakistan to enhance representativeness. The inclusion of objective diagnostic methods, such as upper endoscopy, impedance-pH monitoring, and gastric motility testing, may also yield stronger insights. Additionally, the effects of lifestyle interventions, stress management, and pharmacological methods on these conditions should be investigated in future studies to support evidence-based management.

## Conclusions

The research showed that GERD symptoms and severity of dyspepsia were significantly associated in adults in Pakistan, and gender, age, family history, and chronic illnesses were related to symptom severity. The results highlight the overlap between functional gastrointestinal disorders and the importance of combined diagnostic methods in clinical practice. The symptom burden and quality of life may be improved by addressing modifiable risk factors (i.e., diet, smoking, and stress) and implementing specific treatment plans. These findings require further research to authenticate them and to develop locally specific, holistic management interventions tailored to each region's particular needs.

## Appendices

Appendix A 

**Table 9:** Demographic information questionnnaire.

Items	Responses
Age	-
Gender	-
Marital status	-
Education	-
Occupation	-
Smoking status	-
Family history of GI disorders (GERD/Dyspepsia/Peptic ulcer)	-
Any known chronic illness (multiple choice possible)	-

Appendix B 

**Table 10:** Gastroesophageal Reflux Disease Questionnaire (GERD-Q). Source: Jones et al. (2009) [12].

Items	Response
How often did you have a burning feeling behind your breastbone (heartburn)?	-
How often did you have stomach contents (liquid or food) moving upwards to your throat or mouth (regurgitation)?	-
How often did you have a pain in the centre of the upper stomach?	-
How often did you have nausea?	-
How often did you have difficulty getting a good night’s sleep because of your heartburn and/or regurgitation?	-
How often did you take additional medication for your heartburn and/or regurgitation, other than what the physician told you to take?	-

Appendix C 

**Table 11:** Short-Form Leeds Dyspepsia Questionnaire (SF-LDQ). Source: Fraser et al. (2007) [13].

Items	Responses
Indigestion: Indigestion is a pain or discomfort in the upper abdomen.	-
Heartburn: Heartburn is a burning feeling behind the breastbone.	-
Regurgitation: Regurgitation is an acid taste coming up into your mouth from your stomach.	-
Nausea: Nausea is a feeling of sickness without actually being sick.	-

## References

[1] Maret-Ouda J, Markar SR, Lagergren J (2020). Gastroesophageal reflux disease: a review. JAMA.

[2] Lacy BE, Weiser K, Chertoff J (2010). The diagnosis of gastroesophageal reflux disease. Am J Med.

[3] Malfertheiner P, Hallerbäck B (2005). Clinical manifestations and complications of gastroesophageal reflux disease (GERD). Int J Clin Pract.

[4] El-Serag HB, Sweet S, Winchester CC, Dent J (2014). Update on the epidemiology of gastro-oesophageal reflux disease: a systematic review. Gut.

[5] Eslick GD, Talley NJ (2009). Gastroesophageal reflux disease (GERD): risk factors, and impact on quality of life-a population-based study. J Clin Gastroenterol.

[6] Ford AC, Mahadeva S, Carbone MF, Lacy BE, Talley NJ (2020). Functional dyspepsia. Lancet.

[7] Enck P, Azpiroz F, Boeckxstaens G (2017). Functional dyspepsia. Nat Rev Dis Primers.

[8] Bytzer P, Talley NJ (2001). Dyspepsia. Ann Intern Med.

[9] de Bortoli N, Tolone S, Frazzoni M (2018). Gastroesophageal reflux disease, functional dyspepsia and irritable bowel syndrome: common overlapping gastrointestinal disorders. Ann Gastroenterol.

[10] Gerson LB, Kahrilas PJ, Fass R (2011). Insights into gastroesophageal reflux disease-associated dyspeptic symptoms. Clin Gastroenterol Hepatol.

[11] Iqbal N, Khan A, Yousaf M (2024). Prevalence and risk factors of gastroesophageal reflux disease (GERD) in medical college students. Biol Clin Sci Res J.

[12] Naing L, Nordin RB, Abdul Rahman H, Naing YT (2022). Sample size calculation for prevalence studies using Scalex and ScalaR calculators. BMC Med Res Methodol.

[13] Jones R, Junghard O, Dent J, Vakil N, Halling K, Wernersson B, Lind T (2009). Development of the GerdQ, a tool for the diagnosis and management of gastro-oesophageal reflux disease in primary care. Aliment Pharmacol Ther.

[14] Fraser A, Delaney BC, Ford AC, Qume M, Moayyedi P (2007). The Short-Form Leeds Dyspepsia Questionnaire validation study. Aliment Pharmacol Ther.

[15] Quigley EM, Lacy BE (2013). Overlap of functional dyspepsia and GERD-diagnostic and treatment implications. Nat Rev Gastroenterol Hepatol.

[16] Kim YS, Kim N, Kim GH (2016). Sex and gender differences in gastroesophageal reflux disease. J Neurogastroenterol Motil.

[17] Kim YS, Kim N (2020). Functional dyspepsia: a narrative review with a focus on sex-gender differences. J Neurogastroenterol Motil.

[18] Nilsson M, Johnsen R, Ye W, Hveem K, Lagergren J (2004). Prevalence of gastro-oesophageal reflux symptoms and the influence of age and sex. Scand J Gastroenterol.

[19] Jones R, Lydeard S (1989). Prevalence of symptoms of dyspepsia in the community. BMJ.

[20] Ruderstam H, Ohlsson B (2022). Self-reported IBS and gastrointestinal symptoms in the general population are associated with asthma, drug consumption and a family history of gastrointestinal diseases. Scand J Gastroenterol.

[21] Klausen KM, Bomme Høgh M, David M, Schaffalitzky de Muckadell OB, Hansen JM (2021). How dyspepsia, gastroesophageal reflux symptoms, and overlapping symptoms affect quality of life, use of health care, and medication - a long-term population based cohort study. Scand J Gastroenterol.

[22] Locke GR 3rd, Zinsmeister AR, Talley NJ, Fett SL, Melton LJ 3rd (2000). Familial association in adults with functional gastrointestinal disorders. Mayo Clin Proc.

